# The “IAG-Switch”—A Key Controlling Element in Decapod Crustacean Sex Differentiation

**DOI:** 10.3389/fendo.2020.00651

**Published:** 2020-09-10

**Authors:** Tom Levy, Amir Sagi

**Affiliations:** ^1^Department of Life Sciences, Ben-Gurion University of the Negev, Beer-Sheva, Israel; ^2^The National Institute for Biotechnology in the Negev, Ben-Gurion University of the Negev, Beer-Sheva, Israel

**Keywords:** androgenic gland, IAG-switch, insulin-like androgenic gland hormone, monosex population, sex determination, sex differentiation, sexual plasticity

## Abstract

The androgenic gland (AG)–a unique crustacean endocrine organ that secretes factors such as the insulin-like androgenic gland (IAG) hormone—is a key player in crustacean sex differentiation processes. IAG expression induces masculinization, while the absence of the AG or a deficiency in IAG expression results in feminization. Therefore, by virtue of its universal role as a master regulator of crustacean sexual development, the IAG hormone may be regarded as the sexual “IAG-switch.” The switch functions within an endocrine axis governed by neuropeptides secreted from the eyestalks, and interacts downstream with specific insulin receptors at its target organs. In recent years, IAG hormones have been found—and sequenced—in dozens of decapod crustacean species, including crabs, prawns, crayfish and shrimps, bearing different types of reproductive strategies—from gonochorism, through hermaphroditism and intersexuality, to parthenogenesis. The IAG-switch has thus been the focus of efforts to manipulate sex developmental processes in crustaceans. Most sex manipulations were performed using AG ablation or knock-down of the *IAG* gene in males in order to sex reverse them into “neo-females,” or using AG implantation/injecting AG extracts or cells into females to produce “neo-males.” These manipulations have highlighted the striking crustacean sexual plasticity in different species and have permitted the manifestation of either maleness or femaleness without altering the genotype of the animals. Furthermore, these sex manipulations have not only facilitated fundamental studies of crustacean sexual mechanisms, but have also enabled the development of the first IAG-switch-based monosex population biotechnologies, primarily for aquaculture but also for pest control. Here, we review the crustacean IAG-switch, a unique crustacean endocrine mechanism, from the early discoveries of the AG and the IAG hormone to recent IAG-switch-based manipulations. Moreover, we discuss this unique early pancrustacean insulin-based sexual differentiation control mechanism in contrast to the extensively studied mechanisms in vertebrates, which are based on sex steroids.

## Introduction

To put the subject of this review into context, we start with a brief history of the discovery of the androgenic gland (AG) in crustaceans. In 1947, in the course of an anatomical/histological study of the male reproductive system in the blue swimming crab *Callinectes sapidus*, an “accessory” endocrine gland was found adjacent to the sperm duct (1). Some years later, this gland was termed the “androgenic gland” in light of its key role in crustacean masculine differentiation, as shown by functional experiments of AG ablation and implantation in the amphipod crustacean *Orchestia gammarella* (2). In later experiments, testis removal in males or implanting females with testicular tissues was found to be ineffective in causing sex reversal, thus suggesting that the vertebrate-like gonadal testosterone is probably not involved in crustacean masculine differentiation (3), and indeed, in 1964, it was first reported that the cells of the AG bore greater similarity to vertebrate protein-producing cells than to steroid-producing cells (4). Thereafter, some functional experiments involving the AG were performed not only by AG grafting but also by injections of AG extracts (5). However, it took a while until a specific AG hormone was first isolated from the terrestrial isopod *Armadillidium vulgare* (6, 7). In 2007, subsequent to the first transcriptomic identification of this hormone in a decapod—the redclaw crayfish *Cherax quadricarinatus*—further validation revealed the hormone structure to be that of an insulin-like peptide (ILP) family member, and the hormone was thus termed the “insulin-like androgenic gland” (IAG) hormone (8). It took about another 10 years before the first report appeared of the successful chemical synthesis of an IAG hormone—that of the giant freshwater prawn *Macrobrachium rosenbergii*, a commercially (9, 10) and environmentally (11) important species (12). Since its first discovery, the IAG hormone has been isolated and characterized in twenty-nine decapod species (Table 1), including prawns, shrimp, crayfish, lobsters and crabs, some of which are highly important for the aquaculture industry worldwide (13), and, as this review will show, the IAG-based sex differentiation mechanism is undoubtedly unique in the Pancrustacea, a diverse taxon that contains all crustaceans and hexapods.

**Table 1:** IAG in decapod crustacean species.

**Group**	**Family**	**Species**	**GenBank accession number**
**Prawn**	Palaemonidae	*Macrobrachium rosenbergii*	FJ409645.1
		*Macrobrachium nipponense*	JX962354.1
		*Macrobrachium vollenhovenii*	KJ524578.1
		*Macrobrachium lar*	AB579012.1
		*Palaemon paucidens*	AB588013.1
		*Palaemon pacificus*	AB588014.1
**Lobster**	Palinuridae	*Sagmariasus verreauxi*	KF220491.1
		*Jasus edwardsii*	KF908794.1
**Shrimp**	Penaeidae	*Litopenaeus vannamei*	KX589057.1
		*Fenneropenaeus chinensis*	JQ388277.1
		*Penaeus indicus*	MG022137.1
		*Litopenaeus occidentalis*	KX589058.1
		*Litopenaeus stylirostris*	KX589059.1
		*Marsupenaeus japonicus*	AB598415.1
		*Penaeus monodon*	GU208677.1
	Pandalidae	*Pandalus platyceros*	KX619617.1
**Crab**	Varunidae	*Hemigrapsus sanguineus*	MH580760.1
		*Eriocheir sinensis*	KU724192.1
	Geryonidae	*Chaceon quinquedens*	KY497474.1
	Portunidae	*Portunus pelagicus*	HM459854.1
		*Scylla paramamosain*	JQ681748.1
		*Callinectes sapidus*	HM594945.1
		*Portunus trituberculatus*	MH119940.1
		*Carcinus maenas*	HM594946.1
**Crayfish**	Cambaridae	*Procambarus clarkii*	KT343750.1
		*Procambarus virginalis*	MF405195.1
		*Procambarus fallax*	KX619618.1
	Parastacidae	*Cherax quadricarinatus*	DQ851163.1
		*Cherax destructor*	EU718788.1

## Sex Determination and Sex Differentiation in Crustaceans

In most organisms, sex is determined by chromosomes [i.e., genetic sex determination; GSD (14)] rather than by environmental factors [i.e., environmental sex determination; ESD (15)] (16). The most common GSD systems are the XX/XY and WZ/ZZ systems, in which females are homogametic and males are heterogametic in the former mode of inheritance, and vice versa in the latter (17). With some exceptions (18), most prawn, shrimp and crayfish species bear the WZ/ZZ sex determination system (13, 19–23), while some species of crabs and lobsters bear the XX/XY system (24–27). In the animal kingdom, there are only a few reports of sex-determining genes being associated with the W/Z sex chromosomes. Among them are the W-chromosome-associated *DM-W* gene, which is vital for ovarian development in the African clawed frog *Xenopus laevis* (28), and the Z-chromosome-linked *DMRT1* gene, whose dosage is assumed to control the sex determination process in the chicken *Gallus gallus domesticus* (29). In contrast to the sparse knowledge on the genes associated with the W/Z chromosomes (especially in crustaceans), the male sex-determining genes that are associated with the Y chromosome and that control masculinization in animals bearing the XX/XY system have been well-characterized. Among these genes, most mammals have the well-known *SRY* gene (30). Other examples include the DMY/Dmrt1bY gene, which is associated with the formation of the testis in the medaka fish (31), and the recently discovered *iDMY* gene, which is the male sex-determining factor during embryogenesis in the Eastern spiny lobster Sagmariasus verreauxi (25). To reveal the genetic content of the sex chromosomes, extensive karyotyping of different decapod crustacean species has been performed and published. However, none of the available karyotypes can distinguish between the sex and autosomal chromosomes (32–38). Moreover, while genome sequencing using next generation techniques is common and genomes have been published for several decapod species (39–41), none, except that of *M. rosenbergii* (42), is a phased genome in which a certain scaffold could be attributed to a paternal or maternal origin. Therefore, verified sex-determining factors in decapod species, especially those with WZ/ZZ chromosomal content, are yet to be found.

For species in which sex is determined by sex chromosomes (43), the sex differentiation process starts with the expression of genes responsible for promoting masculinization or feminization during early developmental periods. Many such invertebrate genes have been well-studied, including the *mab-3* gene in the nematode *Caenorhabditis elegans* (44), the *transformer-2* (*tra-2*) gene in pancrustaceans (45), and the *doublesex* (*dsx*) gene in the fruit fly *Drosophila melanogaster*, which has alternative spliced variants yielding different sexes (46). Although the information on such sex differentiating genes in crustaceans remains limited, from the few studies that have been conducted, it is known that *dsx* is expressed in the branchiopod *Daphnia magna* (47), and the *dsx and mab-3 related transcription factor* (*DMRT*) is expressed in the testis of the decapod Eriocheir sinensis, the Chinese mitten crab (48). In *M. rosenbergii*, transcriptomic libraries obtained for different developmental stages—from the embryonic stage (49), through larvae and post-larvae, to adults (50, 51)—appear to contain homolog transcripts of the *dsx, tra-2*, and *DMRT* genes. Moreover, *IAG* silencing in *M. rosenbergii* resulted in significant decrease in the expression of two DMRTs and other sex related genes (52). However, the exact relationship of these genes to the sex differentiation mechanism—if such a relationship does indeed exist—has yet to be found.

Although in the next section we will describe the universal *IAG* gene as a master switch involved in crustacean sex differentiation, it is noteworthy that sex differentiation mechanisms in crustaceans are not only mediated by genes but also disrupted by external factors. For example, elevated bacterial dosage of *Wolbachia* reduces the functionality of insulin receptors in isopods which results in feminization (53). Additionally, some environmental pollutants serve as endocrine disrupting chemicals (EDCs) suggested to affect sex differentiation and sexual development in crustaceans (54). The latter concept was shown in various crustacean species from different orders. In daphnids, the exposure to DES, a synthetic estrogen, induced the development of secondary sexual characters like larger abdominal process in females of *D. magna*, while longer first antennae were observed in males exposed to the androgen androstenedione (55). Moreover, in *D. pulex*, exposure to methoprene, a juvenile hormone analog, yielded all-female broods, while gravid females exposed to 20-hydroxyecdysone, has resulted in all-male broods (56). In the amphipod *Gammarus pulex*, exposure to the xenoestrogen 17α-ethynylestradiol increased the females:males sex ratio (57) and in decapods, heavy metals such as cadmium and copper inhibited ovarian growth in the crabs *Uca pugilator* (58) and *Chasmagnathus granulata* (59), while the xenoestrogen 4-nonylphenol reduced testis weight in the crab *Carcinus maenas* (60). A correlation between endocrine disruptors and crustacean sex differentiation is also exemplified by EDCs discharge in polluted areas that increased the frequency of intersexuality in harpacticoid copepods where intersexuality is extremely rare (61), in amphipods (62) and in decapods (63). These findings raised major concerns regarding the impacts of pollutants on the reproductive success of many crustacean species.

## The IAG-Switch–A Master Sex Controlling Device in Crustaceans

**Figure 1:**
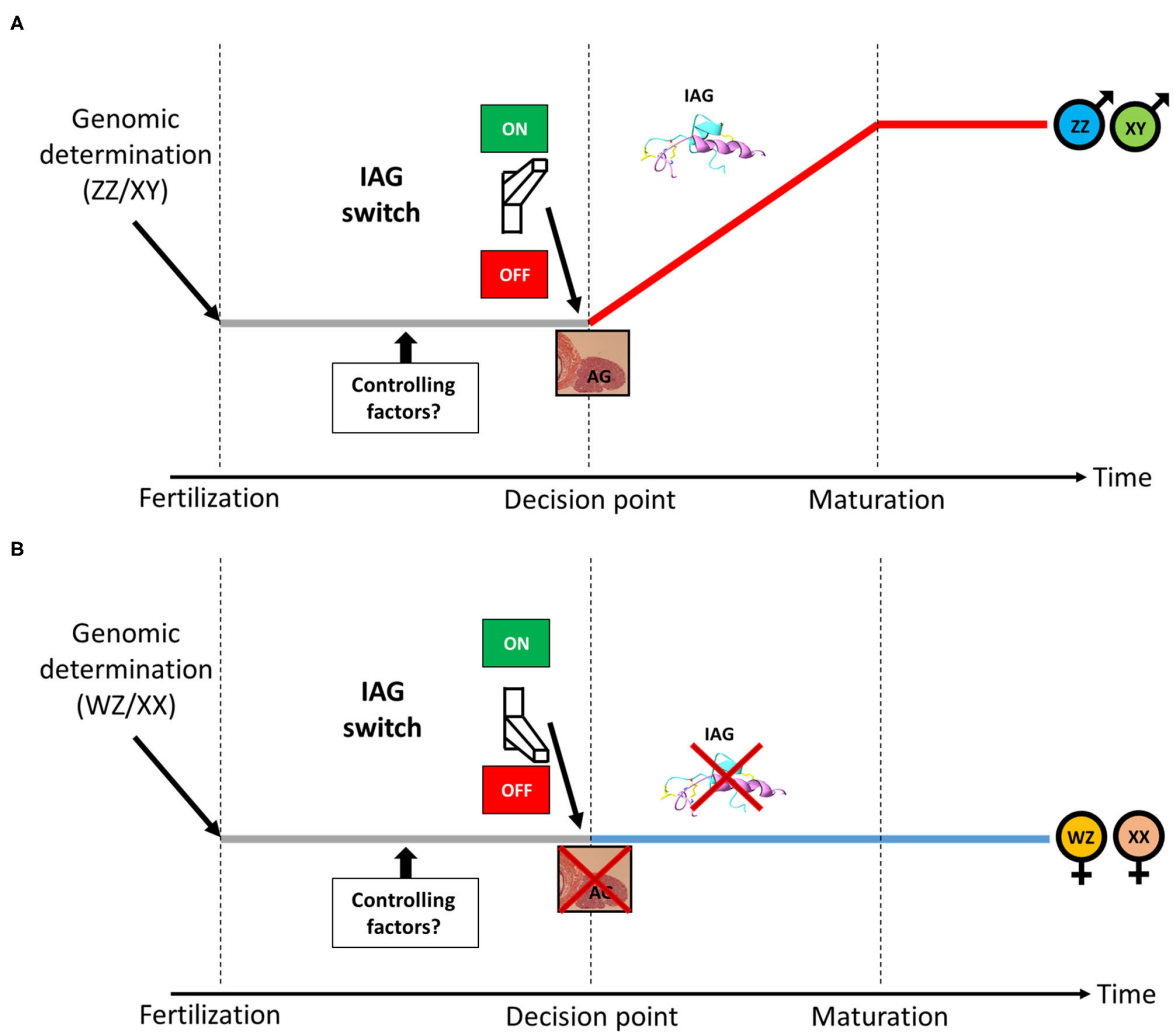
The IAG-switch in gonochoristic crustaceans. **(A)** Following male genotypic determination (ZZ/XY), the IAG-switch initiates the formation of the IAG-secreting AG, leading to the development of a mature male. **(B)** Following female genotypic determination (WZ/XX), the IAG-switch inhibits the formation of the IAG-secreting AG, leading to the development of a mature female. The period in which putative upstream IAG-switch controlling factors are expressed is denoted.

As described above, in male crustaceans, the AG is a unique endocrine organ, secreting the IAG hormone, which serves as a master universal sex-differentiating switch abundant among crustaceans, thus termed the “IAG-switch” (13). A scheme describing the putative location of the IAG-switch from genotypic determination to sexual maturation in gonochoristic crustaceans is given in Figure 1. Residing within the eyestalk-AG-testis endocrine axis (64), the IAG-switch is controlled by upstream neuropeptides and interacts downstream with IAG receptors and binding proteins (51, 65–68). The neuropeptides that mediate growth and reproduction are produced in the X-organ (located in the eyestalk) and later accumulated in the adjacent sinus gland, from where they are secreted. It was found that eyestalk ablation in males caused hypertrophy and hyperplasia of the AG (64, 69) as well as over-expression of the IAG hormone (70) and of a membrane-anchored AG-specific factor (71). Therefore, it was suggested that some X-organ derived neuropeptides are upstream controlling elements of AG activity (64, 72, 73). Moreover, a reduction in the transcript levels of gonad-inhibiting hormone (GIH), molt-inhibiting hormone (MIH) and other eyestalk-derived neuropeptides (by using RNAi knock down) significantly increased IAG expression. On the basis of these findings, it was postulated that these neuropeptides exert an upstream function that controls AG activity (i.e., IAG secretion) (73). It has also been suggested that female molting factors, perceived by males via their short lateral antennules (functioning as olfactory organs), also contribute to the regulation of AG function and male gonadal maturation by increasing IAG expression, thus implying that the IAG-switch is also partially controlled by female reproductive activity (74).

Studying elements that are downstream to the AG within the insulin-like signaling pathway revealed several insulin receptors that interact with the IAG hormone. It was found that some receptors were neither sex specific nor tissue specific, but silencing their encoding genes resulted in AG hypertrophy and over-expression of the IAG hormone (51). In contrast, other receptors were male specific, and their knock down led to the arrest of most of the germ cells in the testes at the secondary spermatocyte stage (vs. those in the control group, which developed into sperm cells) (68). A study of the downstream signaling pathway of the IAG-switch also revealed an insulin-like binding protein (ILBP) that interacted with the IAG hormone, but whose expression was not AG specific (65). This finding indicated that the protein was perhaps synthesized in a location other than the AG, a premise later supported by a study showing that eight ILBPs characterized in a lobster were neither sex specific nor tissue specific (66). Nevertheless, while some studies have demonstrated that eyestalk-derived neuropeptides (e.g., GIH and MIH) are upstream elements to the IAG-switch and insulin receptors are downstream elements, to the best of our knowledge, specific ILBPs associated with the IAG-switch are still to be found.

The pivotal role of the IAG-switch in governing crustacean sexual differentiation has been under study ever since the discovery of the AG and is, in fact, best exemplified by functional experiments that were performed long before the discovery of the IAG. These studies are revisited here to exemplify the pivotal role of the IAG-switch and its universality among crustacean species (see also a summary of IAG-manipulation experiments performed to date in Table 2).

**Table 2:** Summary of experiments that included IAG-switch based manipulations in crustaceans.

**Species**	**Order**	**Method**	**Results**	**References**
*Orchestia gammarellus*	Amphipoda	AG implantation in females	Development of masculine characters and vitellogenesis inhibition	(2, 75)
*Armadillidium vulgare*	Isopoda	AG implantation in females	Transformation of gonads	(76)
*Armadillidium vulgare*	Isopoda	Injection of AG extracts into immature females	Transformation of gonads	(5)
*Carcinus maenus*	Decapoda	AG implantation in females	Development of male secondary characteristics	(77)
*Lysmata seticaudata*	Decapoda	AG implantation in females	Development of male secondary characteristics	(78)
*Pandalus borealis*	Decapoda	AG implantation in females	Development of male secondary characteristics	(79)
*Rhithropanopeus harrisii*	Decapoda	AG implantation in females	Development of male secondary characteristics	(80)
*Palaemon varians*	Decapoda	AG implantation in females	Development of male secondary characteristics	(81, 82)
*Macrobrachium rosenbergii*	Decapoda	AG ablation in males	Loss of masculine appendages and transformation of gonads	(83)
*Macrobrachium rosenbergii*	Decapoda	AG implantation in females	Development of masculine appendages and transformation of gonads	(84)
*Macrobrachium rosenbergii*	Decapoda	AG implantation in females	Full sex reversal of females to males	(20)
*Macrobrachium rosenbergii*	Decapoda	AG ablation in males	Full sex reversal of males to females	(85)
*Macrobrachium rosenbergii*	Decapoda	IAG knock-down using RNAi in males	Full sex reversal of males to females	(86)
*Macrobrachium rosenbergii*	Decapoda	AG cells transplantation in females	Full sex reversal of females to males	(22, 42)
*Procambarus clarkii*	Decapoda	AG implantation in females	Development of male secondary characteristics	(87)
*Procambarus clarkii*	Decapoda	AG implantation in females	Development of masculine characters and inhibition of vitellogenesis	(88)
*Eriocheir japonicus*	Decapoda	AG implantation in females	Development of masculine appendages	(89)
*Cherax destructor*	Decapoda	Injection of AG extracts into females	Development of male gonopores and inhibition of vitellogenesis	(90)
*Cherax quadricarinatus*	Decapoda	AG implantation in females	Development of masculine characters and inhibition of vitellogenesis	(91)
*Cherax quadricarinatus*	Decapoda	AG ablation in male-intersexuals	Loss of male secondary characteristics and induction of vitellogenesis	(92)
*Cherax quadricarinatus*	Decapoda	AG ablation in male-intersexuals	Loss of mating behavior with females and fighting behavior with males	(93)
*Cherax quadricarinatus*	Decapoda	IAG knock-down using RNAi in male-intersexuals	Vitellogenesis induction	(94)
*Scylla paramamosain*	Decapoda	AG implantation in females	Ovarian regression	(95)
*Eriocheir sinensis*	Decapoda	Injection of AG extracts from *S. paramamosain* and *E. sinensis* into females	Development of male gonopods	(96)
*Procambarus virginalis*	Decapoda	AG implantation from *P. clarkii*	Development of male secondary characteristics	(97)
*Litopenaeus vannamei*	Decapoda	AG ablation in males	Loss of masculine appendages and degradation of spermatids in the gonads	(98)
*Litopenaeus vannamei*	Decapoda	AG implantation in females	Partial development of male secondary characteristics	(99)

From an historical point of view, the first researcher to manipulate the IAG-switch, following the discovery of the AG in crustaceans (1), was Charniaux-Cotton (2). In her pioneering experiments on the amphipod *O. gammarellus*, she demonstrated that implantation of the AG into females induced the development of masculine characters and inhibited vitellogenesis, whereas the implantation of testicular tissue had no such effect (2, 3, 75). Later, studies on the terrestrial isopod *A. vulgare* showed that AG implantation (76) or injection of AG extract into females (5) induced partial masculinization, including the transformation of female reproductive organs into testes, sperm ducts and seminal vesicles.

Most IAG-switch manipulation experiments were performed in decapod crustaceans, including shrimp, prawns, crayfish and crabs (100). Among the earliest of such experiments in decapods were those performed on hermaphrodite species (100, 101); for example, AG implantation into females of the simultaneous hermaphrodite, the Monaco shrimp *Lysmata seticaudata* (78), and females of the sequential protandric Northern shrimp *Pandalus borealis* (79) resulted in the development of male secondary characteristics. In gonochoristic species, AG implantation caused episodic development of external male characteristics, as in the green shore crab Carcinus maenus (77) and the Harris mud crab Rhithropanopeus harrisii (80). In the Japanese mitten crab E. japonicus, AG-implanted females developed masculine appendages, even though all of them retained their oviducts (89), but in the mud crab Scylla paramamosain, ovarian regression occurred in female crabs implanted with AG (95). IAG-switch manipulations in crabs also suggested interspecies cross-activity of AG factors, as injection of an AG extract from S. paramamosain or E. sinensis males into E. sinensis females resulted in the development of male gonopods (96).

Many of the studies on the IAG-switch have been conducted in different species of crayfish. In C. quadricarinatus, implantation of hypertrophied AGs into females resulted in the development of masculine secondary sex characters, such as the typical red patch on the chela, and in a significant reduction in the gonadosomatic index and impairment of vitellogenesis (91). Particularly revealing studies were those conducted on intersex C. quadricarinatus animals. In this species, some animals in the population naturally exhibit intersexuality, in which individuals function as males and exhibit male secondary characters, but bear a mix of male and female gonopores and gonads (active testes on one side and a pre-vitellogenic ovary on the other) (102). In AG-ablated C. quadricarinatus intersexuals, there was a partial shift toward femaleness in that external male characters were not regenerated, but expression of the vitellogenin gene was induced (92). Moreover, examination of the agonistic and mating behavior of AG-ablated C. quadricarinatus intersexuals revealed that these animals did not exhibit typical mating behavior when exposed to females or the usual fighting behavior when confronted with males (93). Knocking down the IAG gene—by using RNAi through injection of dsCq-IAG—to C. quadricarinatus intersexuals caused elevation of vitellogenin expression to a level that did not differ from that in intact vitellogenic females (94). In a related species, the common yabby, C. destructor, a significant number of females (vs. a control group) injected with AG extract developed male gonopores at the base of the fifth pereiopod and showed inhibition of vitellogenesis (90). In some early experiments carried out in the red swamp crayfish *Procambarus clarkii*, implantation of AGs into females led to partial masculinization, as shown by the partial transformation of the first pair of pleopods into typical male gonopods (87) and by the inhibition of vitellogenesis in mature females (88). One of the most peculiar IAG-switch-based manipulations was performed on a crayfish species in which males do not exist—the marbled crayfish *P. virginalis*. This parthenogenetic species, in which a virginal form of reproduction gives rise to identical clones of all-female progeny (103, 104), nonetheless expresses the *IAG* gene (105). It was thus suggested that *P. virginalis* is a virginal form that diverged from the gonochoristic slough crayfish *P. fallax* (106), a premise supported by a report that both species retain a highly similar sequence of the *IAG* gene (105). Interestingly, implantation of *P. virginalis* females with AGs from *P. clarkii*, a related species, resulted in the appearance of masculine external characteristics, such as thickening of the first and second pairs of pleopods and the formation of reversed spines on the third and fourth pairs of pleopods (97). The above findings indicate not only that cross-activity of AG factors occurs between species, but surprisingly that in crustacean species, even those in which males do not exist, female animals are still susceptible to the effects of IAG-switch-based manipulations.

Studies on IAG manipulation in species of Palaemonidae have been reported from 1979/80 onwards. Among these, transplantation of AG grafts induced the development of external masculine characteristics in the common ditch shrimp *Palaemon varians* (81, 82), and the first partial sex reversal using IAG-switch manipulation that was undertaken in *M. rosenbergii*, the most extensively studied palaemonid. In the latter study, most andrectomized males did not regenerate the appendix masculina (AM), and a reduction of spermatogenic lobules in the testes was observed, while some AG-ablated males showed the development of female gonopores and oviducts and even the initiation of oogenesis (83). In a complementary study, it was shown that AG-implanted females developed masculine characters, including AMs and sperm ducts, and in some cases spermatogenesis was initiated in the gonads (84). That study also served to highlight the IAG-switch as a pivotal sex differentiating mechanism in crustaceans, since females implanted with sperm duct or testicular tissue were not masculinized, while ~80% of AG-implanted females showed some degree of masculinization (84). In another important aquaculture species, the penaeid Pacific white shrimp *Litopenaeus vannamei*, AG ablation of males in various post-larval stages resulted in inferior development of the AMs and degradation of the spermatids in the gonads (98), while AG implanted females did not develop AMs and only the minority developed male-like claspers on the first endopods (99). However, we note that complete and functional sex reversal in this important species has not yet been achieved, despite the extensive attempts of various research groups around the world.

While the above studies have indeed demonstrated the crucial role of the IAG-switch in sexual differentiation in several orders and many species in the Crustacea, all the above-described cases of IAG-switch manipulations yielded various types of partial sex shifts but not fully functional sex reversal of one sex into the other. As shown in Figure 1, we assume that a putative decision point exists, in which an individual commences toward sexual maturation as a male or a female. It is hypothesized that IAG-switch controlling factors are accommodating this decision point. Functional experiments manipulating such factors will open a new window into IAG-switch upstream controlling mechanism and might achieve a complete shift between sexes. In order to find such IAG-switch controlling factors, advanced next generation sequencing (NGS) techniques may be employed to sequence the RNA of males and females at early developmental stages whose investigation might yield sexually biased genes that putatively control the IAG-switch. Those genes could be manipulated by knockdown techniques such as RNAi (107) or Morpholino oligos (108) and, if performed before the decision point, might lead to a functional shift between sexes. However, timing the decision point is species-specific and body size of the animal in such early developmental stage might be very small which makes the RNAi/Morpholino manipulation complicated. To overcome the size obstacle, a whole genome sequencing of the animal using latest NGS platforms could be used followed by CRISPR-Cas9 genome editing operations (109) that could be performed at the embryonic level and guarantee that the manipulation occurs before the decision point. To the best of our knowledge, CRISPR editing of IAG-switch related factors was never performed. In the next section, successful IAG-switch manipulation resulting with full sex reversal will be described.

## IAG-Switch Based Biotechnologies for Producing Monosex Populations

The use of monosex populations is common in animal husbandry, since in many species males and females yield different agricultural products, particularly fish (110–112), poultry (113–115), and mammals (116). In crustaceans, monosex populations offer particular advantages in aquaculture (85, 117–119), since most decapod species exhibit dimorphic growth patterns, leading to variations in animal size at harvest. The dimorphic growth patterns, in turn, could be a result of different growth rates and behavioral patterns (120) and different food conversion ratio (FCR) values between the sexes (121, 122), or even cannibalism (96). Monosex populations of crustaceans can also be exploited in ecological applications, e.g., monosex prawn populations could be used as bio-control agents, serving as predators of the snails that damage rice crops (123) and that are vectors of parasites hazardous to humans (11, 124–127) and fish (128). Here, we should remember that introducing new species as bio-control agents into a given niche may result in devastating consequences to the ecosystem (129), and therefore monosex populations are preferable as biocontrol agents, since they are not able to reproduce and thus become invasive species.

In the exploitation of monosex aquaculture for yield improvement in crustacean species, the choice of sex will generally be guided by the optimal growth rates and size at harvest. Therefore, all-male aquaculture was proposed for species exhibiting male superiority, as is the case for most crayfish (130–132), lobsters (133), prawns (117, 120, 134) and crabs (135), while all-female aquaculture was suggested for shrimp species in which females are larger than males (118, 136). However, growth rates and size are not the only considerations in the choice of sex for monosex cultures; an additional consideration is the desired product: For example, for the edible female gonads of *E. sinensis* (137), the harvested animals would be vitellogenic females with developed ovaries, even if their body size is smaller than males. Additionally, even in some species in which males are larger than females, such as *M. rosenbergii* prawns (85), monosex female culture could improve the yield and profit in two possible ways: intensification of stocking densities permitted by the lack of aggressiveness of the females (138), and elimination of the need for costly size-selective harvests by virtue of the size uniformity of females (117, 139–141).

**Figure 2:**
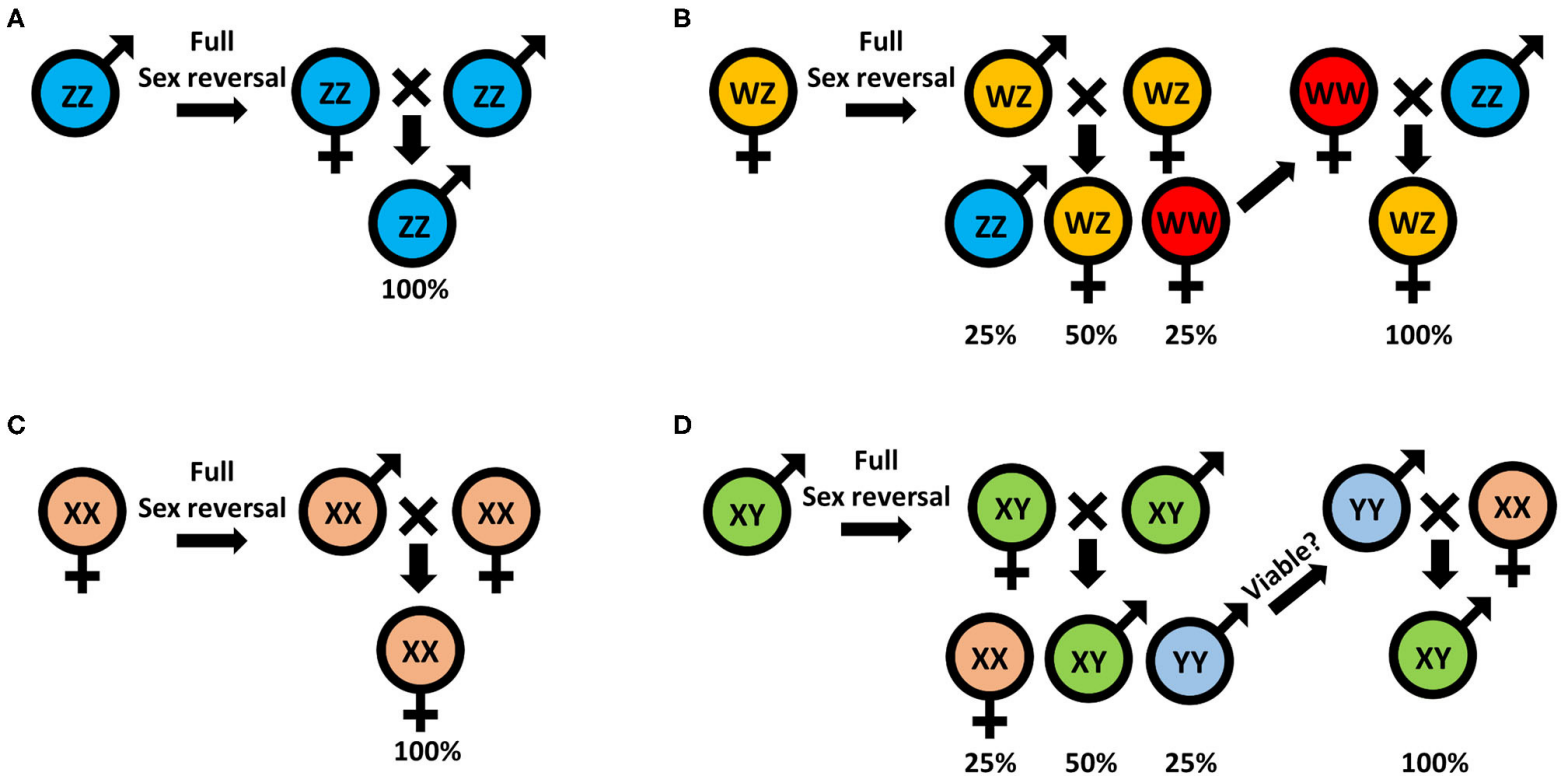
Sex manipulation schemes for monosex populations: **(A)** all-male and **(B)** all-female in animals with the WZ/ZZ genotypic mode of inheritance; and **(C)** all-female and **(D)** all-male in animals with XX/XY genotypic mode of inheritance.

**Figure 3:**
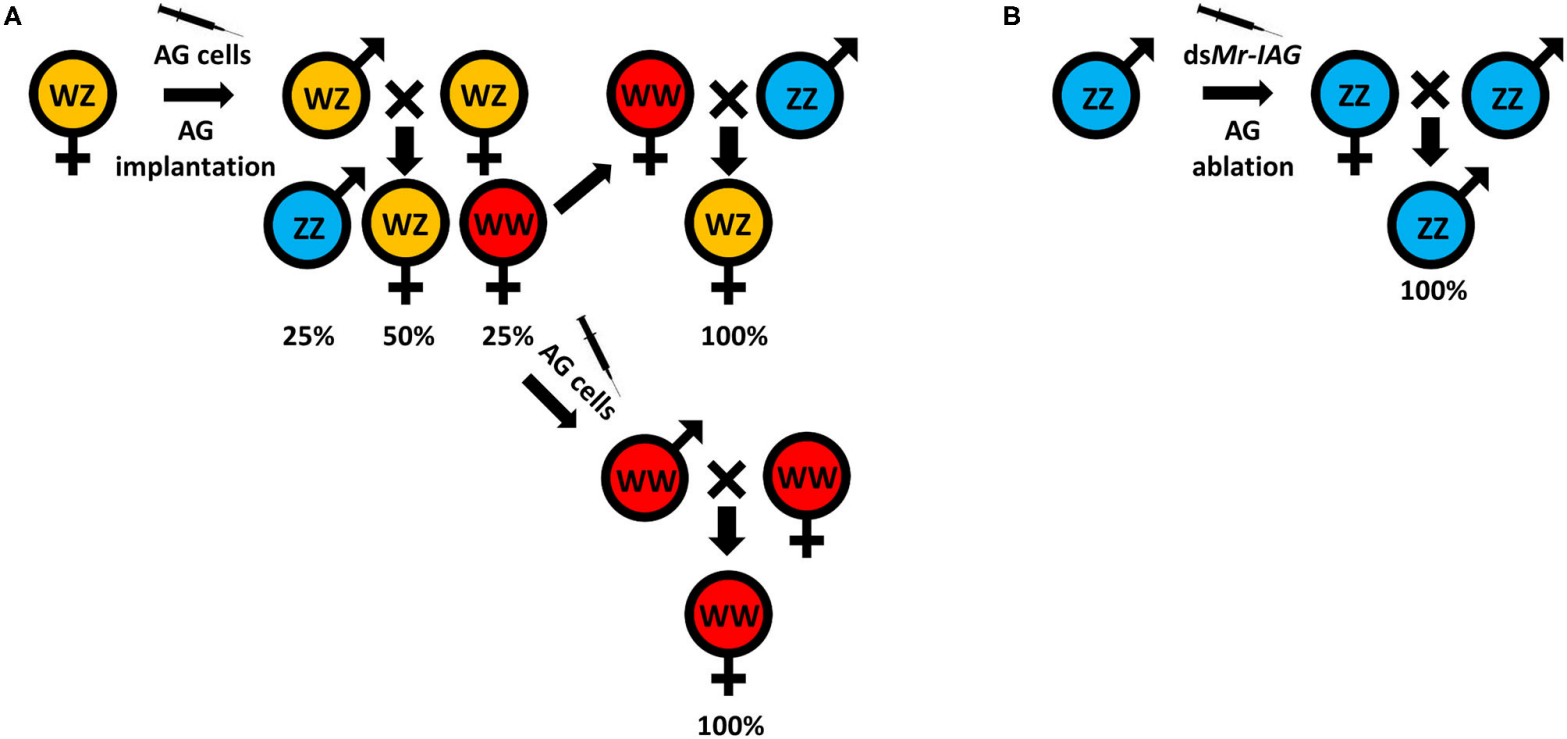
Successful sex manipulations in *M. rosenbergii*. **(A)** A WZ female implanted with an AG (20) or injected with an AG cell suspension (22) inverted into a WZ “neo-male.” The progeny, when crossed with normal WZ females, yielded 25% of WW females. These WW females can be crossed with normal ZZ males to produce all-female WZ populations, or they can be injected with an AG cell suspension (42) to produce WW neo-males that, when crossed with WW females, will give rise to all-female WW populations. **(B)** ZZ males that are AG ablated (85) or injected with ds*Mr-IAG* (86) inverted into ZZ “neo-females” that, when crossed with normal ZZ males, give rise to all-male ZZ populations.

Traditionally, monosex aquaculture is achieved through manual sorting (117, 142), which is both time consuming and labor intensive and does not guarantee a 100% monosex population. Agro-biotechnologies are thus needed to replace this traditional method. To date, efforts to establish either all-male or all-female populations, for both WZ/ZZ and XX/XY sex heritability schemes, start with an initial sex reversal step of male to a female or vice versa, based on manipulating the IAG-switch during the sex differentiation process (see schemes in Figure 2 which represent the methodology to achieve all-male and all-female populations in both WZ/ZZ and XX/XY systems). However, all the IAG-switch manipulations performed to date have resulted only in partial sexual shifts, with the exception of the fully functional sex reversal in the decapod species *M. rosenbergii* (20, 22, 42, 85, 86). All-female progenies of *M. rosenbergii* were achieved in the following way: Implantation of AGs in juvenile WZ females resulted in sex reversal to WZ “neo-males.” When these neo-males were crossed with normal WZ females, a quarter of the progeny comprised viable WW females. Crossing of the WW females with normal ZZ males produced a monosex WZ female population (20) (Figure 3A). In contrast, to produce a monosex ZZ male population, the first step was AG ablation of juvenile ZZ males, which sex reversed them into ZZ “neo-females.” Crossing these neo-females with normal ZZ males produced a monosex ZZ male population (85) (Figure 3B). Even though a single sex reversed animal may yield several monosex progenies of thousands of prawns, the complicated surgical procedure of AG ablation/implantation resulted in high mortality and low rates of fully sex reversed animals (20, 85), and the above sex reversal schemes were therefore not suitable for scaling up toward commercialization. The break-through was made with the development of the first RNAi-based biotechnology for *M. rosenbergii* monosex aquaculture, which relied on knock down of the *IAG* gene through a single injection of ds*Mr-IAG* into ZZ males at an early post-larval stage. This biotechnology successfully enabled mass production of ZZ neo-females and consequently of all-male aquaculture (86) (Figure 3B). This procedure has been commercialized and has already yielded several consecutive generations of W-free ZZ prawns (143).

For generating all-female populations, the complicated procedure of AG implantation into females was replaced with a single injection of AG cell suspension into WZ females at an early post-larval stage; this yielded WZ neo-males and subsequently WW females and all-female aquaculture (22) (Figure 3A). This biotechnology was later improved by using the same procedure of AG cell transplantation into WW females that were then sex reversed into WW neo-males. Crossing of the WW neo-males with WW females yielded all-female WW progenies, thereby making the production of all-female producing females much more efficient (42). We note that when this procedure was performed repeatedly, it, too, yielded consecutive generations of Z-free WW prawns (144).

The above studies on *M. rosenbergii* yielded both males and females of every possible genotype (ZZ, WZ and WW). This sexual plasticity further highlights the pivotal role of the IAG-switch in crustacean sexual differentiation, since even after the genotype is determined, manipulating the IAG-switch may alter the initial direction of sexual development toward maleness or femaleness, with a probable complete autosomal sex-differentiation toolkit for each gender, regardless of the presence or absence of either sex chromosome.

## Why Insulin-Like?

The above review of the pivotal involvement of an ILP as a major factor in crustacean sex differentiation demands a broader evolutionary discussion of ILPs in the context of sex regulation and reproduction in the animal kingdom, in which certain aspects of sexual differentiation are largely controlled by vertebrate-like sex steroids rather than ILPs. Indeed, similar to the IAG hormone, sex steroids (including androgens, estrogens and progestogens) mediate sexual development, secondary sex characters and dimorphic male/female physiological and behavioral patterns (145, 146) in most vertebrates, including mammals, birds, reptiles, amphibians and fish (147). In aquaculture, this role for sex steroids finds application in the production of monosex fish populations by sex reversal of females into males or vice versa through 17α-methyltestosterone or estradiol-17β administration, as applicable (148–150).

An evolutionary survey of the animal kingdom reveals that vertebrate-type sex steroids (mostly estrogen, androgen and progestogen) and their related receptors and binding proteins are also found in aquatic invertebrates, including different classes of mollusks (151) [such as gastropods (152, 153), bivalves (154, 155), and cephalopods (156–158)] and echinoderms (159) [such as starfish (160) and sea urchins (161)]. Moreover, sex steroids are also found in flatworms (162), annelids (163), crustaceans (164), and cnidarians, such as corals (165). Their function in aquatic invertebrates is believed to be associated with reproduction (but not necessarily with sex differentiation) through the control of the levels of noradrenaline and dopamine, gonadic serotonin and catecholamine, and even cell metabolism and immunity (151). Moreover, as is the case for vertebrates (166), in invertebrates sex steroids are involved in growth processes. In crustaceans, and other arthropods, such growth processes depend on a periodic molt cycle in which the animal sheds its old extracellular cuticle and forms a new, larger cuticle (167). In crustaceans, steroid hormones (ecdysteroids) play a major role in molting and other developmental processes that are regulated by neuropeptides, such as MIH secreted from the Y-organ (168–170). Additionally, in some crustaceans, reproduction is linked to a pre-mating molt, which suggests some sort of coordination between molt-controlling steroid agents and gonad maturation (171). However, while the involvement of steroids in growth processes of crustaceans and other arthropods is clear, their function in controlling reproduction in crustaceans has been called into question. Nevertheless, it has been reported that vertebrate-like sex steroids could be involved in crustacean reproduction, as, for example, administration of progesterone induced ovarian maturation and spawning in the shrimp *Metapenaeus ensis* (172) and vitellogenesis in the shrimp *Penaeus japonicus* (173), while estradiol treatments promoted vitellogenesis in the crab *Portunus trituberculatus* (174). In contrast, administration of testosterone to female *Ocypoda platytarsis* crabs resulted in masculinization of the ovaries (175), and when administered to male *Parapenaeopsis hardwickii* shrimp, it even caused hypertrophy and hyperplasia of the AG (176). Moreover, administration of estradiol to entire populations of the freshwater amphipod *G. pulex* and of the decapod *L. vannamei* resulted in a clear female bias (57, 177). However, to the best of our knowledge, there are no reports of a fully functional sex reversal in crustaceans following the administration of vertebrate-like sex steroids, which implies that a different factor might be the main regulator in crustacean sexual differentiation. As described above, it is likely that an ILP, namely, the IAG hormone, is such a controlling element. Nonetheless, questions regarding the evolvement of an insulin-like factor, rather than a steroid, as the master sex controlling switch in crustaceans, and possible interactions between ILPs and steroids remain open.

As a step toward addressing these questions, let us examine the insulin superfamily. This group of proteins includes ILPs with a typical proteomic structure of B and A chains linked by disulfide bonds (178). Peptides of the insulin family are found in protozoans (179) and metazoans—both vertebrates and invertebrates (180). ILPs were first discovered in mammals and attracted extensive interest due to their involvement in many physiological processes (181). In vertebrates, they comprise a set of proteins including insulin, insulin-like growth factors (IGFs) and relaxins, which are essential in reproduction, growth, and developmental and metabolic pathways, such as carbohydrate and lipid metabolism (182–185).

In invertebrates, the first ILP was found in the clam *Mya arenaria* (186), and since then such proteins have been found in many species across different classes, including mollusks (187), annelids (188), flatworms (189), cnidarians (190), sponges (191), nematodes (192) and arthropods (193). A regulatory interaction between ILPs and steroids has indeed been found in insects in which the prothoracicotropic hormone (PTTH), a brain neuropeptide, controls the secretion of the ecdysteroids that regulate molting (194). Bombyxin, such a PTTH found in the silkworm *Bombyx mori* was found to be homologous to insulin (195). In addition to its affect on growth (196) and cell proliferation (197) in lepidopterans, bombyxin is also involved in ovarian development in dipterans (198). ILPs are also found in orthopterans, such as the migratory locust *Locusta migratoria*, in which a single copy of an ILP is expressed as two transcripts; one serving as a putative neurohormone is expressed in the brain, and the other serving as a putative growth factor is not tissue specific (199). In addition to their structural resemblance at the protein level, the conservation of invertebrate ILPs within vertebrates is best exemplified by the fact that an insulin-like protein extracted from the common fruit fly, *D. melanogaster*, showed cross reactivity between species by initiating insulin bioactivity in mice (200), while mammalian insulin was successful in activating *D. melanogaster* insulin receptors (201). Moreover, injection of recombinant human insulin into the shrimp *L. vannamei* led to increased levels of glucose in the hemolymph and of glycogen in the gills, thus suggesting that ILPs play a role in crustacean carbohydrate metabolism (202). Crustaceans are also known to possess ILPs that serve as growth factors (65) and some that regulate glucose metabolism and participate in the immune response against pathogens (203). However, while ILPs are generally not regarded as sex specific, crustaceans constitute a unique group in which a male-specific ILP (the IAG hormone) is the master factor in regulating sexual differentiation (8). This function of an ILP raises questions regarding the speciation of insulins into a variety of different physiological pathways during evolution, especially during the shift from invertebrates to vertebrates. However, while crustacean ILPs and vertebrate-like steroids share a physiological function as growth factors, the evolutionary processes regarding the alteration of the major sex differentiating mechanism from ILPs in early pancrustaceans (i.e., the crustacean IAG-switch) to sex steroids in vertebrates are still unknown.

## Conclusions

The process of crustacean sexual development from the genotypic sex determination (WZ, ZZ, XX or XY), through the sexual differentiation process, to the final masculine or feminine maturation involves various sex controlling mechanisms that include factors such as ILPs or steroids. The IAG-switch is a unique crustacean endocrine-controlling mechanism involving an ILP that regulates sexual differentiation and function within the eyestalk-AG-testis endocrine axis. Despite earlier determination of the sexual genotype, the switch can be manipulated to induce either masculinization or feminization, thereby revealing striking sexual plasticity in crustaceans. It is this sexual plasticity that is often being exploited for sex manipulations for the establishment of monosex populations.

During the evolution of ILPs in the animal kingdom, numerous functions have evolved for such proteins in both invertebrates and vertebrates. A unique ILP function that evolved in the Crustacea is the IAG-switch mechanism, which constitutes the pivotal element in the sex differentiation processes. Nevertheless, to reveal the evolutionary pathways of sex differentiating controllers, i.e., ILPs in crustaceans and sex steroids in vertebrates, further evolutionary studies focusing on sex differentiation during the evolution of arthropods and the shift from invertebrates to vertebrates are required.

## Author Contributions

This manuscript was conceived and written by TL and AS. All authors contributed to the article and approved the submitted version.

## Conflict of Interest

The authors declare that the research was conducted in the absence of any commercial or financial relationships that could be construed as a potential conflict of interest.

## References

[1] Cronin LE. Anatomy and histology of the male reproductive system of *Callinectes sapidus* Rathbun. J Morphol. (1947) 81:209–39. 10.1002/jmor.1050810205 20258886

[2] Charniaux-Cotton H Étude du déterminisme des caractères sexuels secondaires par castration chirurgicale et implantation d'ovaire chez un Crustacé Amphipode (*Orchestia gammarella*). Comptes Rend Acad des Sci Paris. (1953) 236:141–3. 13033322

[3] Charniaux-Cotton H. Androgenic gland of crustaceans. General Comparative Endocrinol Suppl. (1962) 1:241–7. 10.1016/0016-6480(62)90095-3 13878306

[4] King DS. Fine structure of the androgenic gland of the crab, *Pachygrapsus crassipes*. Gen Comp Endocrinol. (1964) 4:533–44. 10.1016/0016-6480(64)90062-0 14216261

[5] Katakura Y Hasegawa Y. Masculinization of females of the isopod crustacean, *Armadillidium vulgare*, following injections of an active extract of the androgenic gland. Gen Comp Endocrinol. (1983) 49:57–62. 10.1016/0016-6480(83)90007-2 6826049

[6] Hasegawa Y Haino-Fukushima K Katakura Y. Isolation and properties of androgenic gland hormone from the terrestrial isopod, *Armadillidium vulgare*. Gen Comp Endocrinol. (1987) 67:101–10. 10.1016/0016-6480(87)90209-7 3623064

[7] Martin G Sorokine O Moniatte M Bulet P Hetru C Van Dorsselaer A. The structure of a glycosylated protein hormone responsible for sex determination in the isopod, *Armadillidium vulgare*. Eur J Biochem. (1999) 262:727–36. 10.1046/j.1432-1327.1999.00442.x 10411634

[8] Manor R Weil S Oren S Glazer L Aflalo ED Ventura T. Insulin and gender: an insulin-like gene expressed exclusively in the androgenic gland of the male crayfish. Gen Comp Endocrinol. (2007) 150:326–36. 10.1016/j.ygcen.2006.09.006 17094989

[9] New MB Freshwater Prawns: status of global aquaculture, 1987. NACA Technical Manual No. 6. In: A World Food Day Publication of the Network of Aquaculture Centers in Asia. Bangkok (1988).

[10] New MB Status of freshwater prawn farming: a review. Aquac Res. (1995) 26:1–54. 10.1111/j.1365-2109.1995.tb00859.x

[11] Savaya-Alkalay A Ovadia O Barki A Sagi A Size-selective predation by all-male prawns: implications for sustainable biocontrol of snail invasions. Biol Invasions. (2018) 20:137–49. 10.1007/s10530-017-1522-1

[12] Katayama H Nagasawa H. Chemical synthesis of N-glycosylated insulin-like androgenic gland factor from the freshwater prawn *Macrobrachium rosenbergii*. J Peptide Sci. (2019) 25:e3215. 10.1002/psc.3215 31515898

[13] Levy T Aflalo ED Sagi A Sex control in cultured decapod crustaceans. In: Sex Control in Aquaculture. Hoboken, NJ: John Wiley & Sons, Inc. (2018). p. 689–704. 10.1002/9781119127291.ch35

[14] Hake L O'Connor C Genetic mechanisms of sex determination. Nat Educ. (2008) 1:25.

[15] Adams J Greenwood P Naylor C Evolutionary aspects of environmental sex determination. Int J Invertebrate Reproduction Dev. (1987) 11:123–35. 10.1080/01688170.1987.10510273

[16] Ashman TL Bachtrog D Blackmon H Goldberg EE Hahn MW Kirkpatrick M. Tree of sex: a database of sexual systems. Sci Data. (2014) 1:140015. 10.1038/sdata.2014.15 25977773 PMC4322564

[17] Gamble T Zarkower D. Sex determination. Curr Biol. (2012) 22:R257–62. 10.1016/j.cub.2012.02.054 22537624 PMC11003734

[18] Waiho K Shi X Fazhan H Li SK Zhang YL Zheng HP. High-density genetic linkage maps provide novel insights into ZW/ZZ sex determination system and growth performance in mud Crab (*Scylla paramamosain*). Front Genet. (2019) 10:298. 10.3389/fgene.2019.00298 31024620 PMC6459939

[19] Katakura Y Endocrine and genetic control of sex differentiation in the malacostracan Crustacea. Invertebr Reprod Dev. (1989) 16:177–82. 10.1080/07924259.1989.9672075

[20] Malecha SR Nevin PA Ha P Barck LE Lamadridrose Y Masuno S Sex-ratios and sex-determination in progeny from crosses of surgically sex-reversed freshwater prawns, *Macrobrachium rosenbergii*. Aquaculture. (1992) 105:201–18. 10.1016/0044-8486(92)90087-2

[21] Parnes S Khalaila I Hulata G Sagi A. Sex determination in crayfish: are intersex *Cherax quadricarinatus* (Decapoda, Parastacidae) genetically females? Genet Res. (2003) 82:107–16. 10.1017/S0016672303006372 14768895

[22] Levy T Rosen O Eilam B Azulay D Aflalo ED Manor R. A single injection of hypertrophied androgenic gland cells produces all-female aquaculture. Marine Biotechnol. (2016) 18:554–63. 10.1007/s10126-016-9717-5 27650072

[23] Yu Y Zhang XJ Yuan JB Wang QC Li SH Huang H. Identification of sex-determining loci in Pacific white shrimp *Litopeneaus vannamei* using linkage and association analysis. Marine Biotechnol. (2017) 19:277–86. 10.1007/s10126-017-9749-5 28508952

[24] Niiyama H The XY chromosomes of the shore-crab, *Hemigrapsus sanguineus* (de Haan). Japanese J Genetics. (1938) 14:34–8. 10.1266/jjg.14.34

[25] Chandler JC Fitzgibbon QP Smith G Elizur A Ventura T. Y-linked iDmrt1 paralogue (iDMY) in the Eastern spiny lobster, *Sagmariasus verreauxi*: the first invertebrate sex-linked Dmrt. Dev Biol. (2017) 430:337–45. 10.1016/j.ydbio.2017.08.031 28864068

[26] Lv JJ Sun DF Huan PP Song L Liu P Li J. QTL mapping and marker identification for sex-determining: indicating XY sex determination system in the swimming crab (*Portunus trituberculatus*). Front Genetics. (2018) 9:337. 10.3389/fgene.2018.00337 30210528 PMC6119780

[27] Fang S Zhang Y Shi X Zheng H Li S Zhang Y. Identification of male-specific SNP markers and development of PCR-based genetic sex identification technique in crucifix crab (*Charybdis feriatus*) with implication of an XX/XY sex determination system. Genomics. (2019) 112:404–11. 10.1016/j.ygeno.2019.03.003 30851358

[28] Yoshimoto S Okada E Umemoto H Tamura K Uno Y Nishida-Umehara C. A W-linked DM-domain gene, DM-W, participates in primary ovary development in *Xenopus laevis*. Proc Natl Acad Sci USA. (2008) 105:2469–74. 10.1073/pnas.0712244105 18268317 PMC2268160

[29] Smith CA Roeszler KN Ohnesorg T Cummins DM Farlie PG Doran TJ. The avian Z-linked gene *DMRT1* is required for male sex determination in the chicken. Nature. (2009) 461:267. 10.1038/nature08298 19710650

[30] Goodfellow PN Lovell-Badge R SRY and sex determination in mammals. Annu Rev Genet. (1993) 27:71–92. 10.1146/annurev.ge.27.120193.000443 8122913

[31] Matsuda M Nagahama Y Shinomiya A Sato T Matsuda C Kobayashi T. DMY is a Y-specific DM-domain gene required for male development in the medaka fish. Nature. (2002) 417:559. 10.1038/nature751 12037570

[32] Mittal OP Dhall U Chromosome studies in three species of freshwater decapods (Crustacea). Cytologia. (1971) 36:633 10.1508/cytologia.36.633

[33] Damrongphol P Eangchuan N Ajpru S Poolsanguan B Withyachumnarnkul B Karyotype of the giant freshwater prawn, *Macrobrachium rosenbergii*. J Sci Soc Thailand. (1991) 17:57–69. 10.2306/scienceasia1513-1874.1991.17.057

[34] Justo CC Murofushi M Aida K Hanyu I Karyological studies on the freshwater prawn *Macrobrachium rosenbergii*. Aquaculture. (1991) 97:327–34. 10.1016/0044-8486(91)90325-2

[35] Campos Ramos R Chromosome studies on the marine shrimps *Penaeus vannamei* and *P. californiensis (Decapoda)*. J Crustacean Biol. (1997) 17:666–73. 10.1163/193724097X00099

[36] Morelli M Le Dean L Vonau V Diter A Karyotype of the marine shrimp *Penaeus indicus* (Crustacea, Decapoda) established by using an image analysis system. Ophelia. (1998) 49:83–95. 10.1080/00785326.1998.10409375

[37] Zhu D Wang C Li ZQ Karyotype analysis on *Portunus trituberculatus*. J Fish China. (2005) 5:649–53.

[38] Scalici M Solano E Gibertini G Karyological analyses on the Australian crayfish *Cherax destructor* (Decapoda: Parastacidae). J Crustacean Biol. (2010) 30:528–30. 10.1651/09-3200.1

[39] Kenny NJ Sin YW Shen X Zhe Q Wang W Chan TF. Genomic sequence and experimental tractability of a new decapod shrimp model, *Neocaridina denticulata*. Mar Drugs. (2014) 12:1419–37. 10.3390/md12031419 24619275 PMC3967219

[40] Gutekunst J Andriantsoa R Falckenhayn C Hanna K Stein W Rasamy J. Clonal genome evolution and rapid invasive spread of the marbled crayfish. Nat Ecol Evol. (2018) 2:567. 10.1038/s41559-018-0467-9 29403072

[41] Zhang X Yuan J Sun Y Li S Gao Y Yu Y. Penaeid shrimp genome provides insights into benthic adaptation and frequent molting. Nat Commun. (2019) 10:356. 10.1038/s41467-018-08197-4 30664654 PMC6341167

[42] Levy T Rosen O Manor R Dotan S Azulay D Abramov A. Production of WW males lacking the masculine Z chromosome and mining the *Macrobrachium rosenbergii* genome for sex-chromosomes. Sci Rep. (2019) 9:1–11. 10.1038/s41598-019-47509-6 31455815 PMC6712010

[43] Janzen FJ Phillips PC. Exploring the evolution of environmental sex determination, especially in reptiles. J Evol Biol. (2006) 19:1775–84. 10.1111/j.1420-9101.2006.01138.x 17040374

[44] Shen MM Hodgkin J. Mab-3, a gene required for sex-specific yolk protein expression and a male-specific lineage in C. elegans. Cell. (1988) 54:1019–31. 10.1016/0092-8674(88)90117-1 3046751

[45] Belote JM Baker BS. Sex determination in *Drosophila melanogaster*- analysis of transformer-2, a sex-transforming locus. Proc Natl Acad Sci USA. (1982) 79:1568–72. 10.1073/pnas.79.5.1568 6803244 PMC346016

[46] Burtis KC Baker BS. *Drosophila* doublesex gene controls somatic sexual-differentiation by producing alternatively spliced messenger-RNAs encoding related sex-specific polypeptides. Cell. (1989) 56:997–1010. 10.1016/0092-8674(89)90633-8 2493994

[47] Kato Y Kobayashi K Watanabe H Iguchi T. Environmental sex determination in the branchiopod crustacean *Daphnia magna*: deep conservation of a doublesex gene in the sex-determining pathway. PLoS Genet. (2011) 7:e1001345. 10.1371/journal.pgen.1001345 21455482 PMC3063754

[48] Zhang EF Qiu GF. A novel Dmrt gene is specifically expressed in the testis of Chinese mitten crab, *Eriocheir sinensis*. Dev Genes Evol. (2010) 220:151–9. 10.1007/s00427-010-0336-2 20809137

[49] Amterat Abu Abayed F Manor R Aflalo ED Sagi A. Screening for *Dmrt* genes from embryo to mature *Macrobrachium rosenbergii* prawns. Gen Comp Endocrinol. (2019) 282:113205. 10.1016/j.ygcen.2019.06.009 31201800

[50] Ventura T Manor R Aflalo ED Chalifa-Caspi V Weil S Sharabi O. Post-embryonic transcriptomes of the prawn *Macrobrachium rosenbergii:* Multigenic succession through metamorphosis. PLoS ONE. (2013) 8:55322. 10.1371/journal.pone.0055322 23372848 PMC3555924

[51] Sharabi O Manor R Weil S Aflalo ED Lezer Y Levy T. Identification and characterization of an insulin-like receptor involved in crustacean reproduction. Endocrinology. (2015) 157:928–41. 10.1210/en.2015-1391 26677879

[52] Tan K Zhou M Jiang H Jiang D Li Y Wang W. siRNA-mediated *MrIAG* silencing induces sex reversal in *Macrobrachium rosenbergii*. Marine Biotechnol. (2020) 22:456–66. 10.1007/s10126-020-09965-4 32337657

[53] Herran B Geniez S Delaunay C Raimond M Lesobre J Bertaux J. The shutting down of the insulin pathway: a developmental window for *Wolbachia* load and feminization. Sci Rep. (2020) 10:10551. 10.1038/s41598-020-67428-1 32601334 PMC7324399

[54] Rodriguez EM Medesani DA Fingerman M. Endocrine disruption in crustaceans due to pollutants: a review. Comp Biochem Physiol A, Mol Integr Physiol. (2007) 146:661–71. 10.1016/j.cbpa.2006.04.030 16753320

[55] Olmstead AW LeBlanc GA Effects of endocrine-active chemicals on the development of sex characteristics of *Daphnia magna*. Environ Toxicol Chem. (2000) 19:2107–13. 10.1002/etc.5620190821

[56] Peterson JK Kashian DR Dodson SI. Methoprene and 20-OH-ecdysone affect male production in *Daphnia pulex*. Environ Toxicol Chem. (2001) 20:582–8. 10.1002/etc.5620200318 11349860

[57] Watts MM Pascoe D Carroll K. Population responses of the freshwater amphipod *Gammarus pulex* (L.) to an environmental estrogen, 17alpha-ethinylestradiol. Environ Toxicol Chem. (2002) 21:445–50. 10.1002/etc.5620210230 11833814

[58] Rodriguez EM Lopez Greco LS Fingerman M. Inhibition of ovarian growth by cadmium in the fiddler crab, *Uca pugilator* (Decapoda, ocypodidae). Ecotoxicol Environ Saf. (2000) 46:202–6. 10.1006/eesa.1999.1896 10831334

[59] Medesani DA Lopez Greco LS Rodriguez EM. Interference of cadmium and copper with the endocrine control of ovarian growth, in the estuarine crab *Chasmagnathus granulata*. Aquatic Toxicol. (2004) 69:165–74. 10.1016/j.aquatox.2004.05.003 15261452

[60] Lye CM Bentley MG Galloway T. Effects of 4-nonylphenol on the endocrine system of the shore crab, *Carcinus maenas*. Environ Toxicol. (2008) 23:309–18. 10.1002/tox.20344 18214899

[61] Moore CG Stevenson JM The occurrence of intersexuality in harpacticoid copepods and its relationship with pollution. Mar Pollut Bull. (1991) 22:72–4. 10.1016/0025-326X(91)90139-J

[62] Ford AT Fernandes TF Rider SA Read PA Robinson CD Davies IM. Endocrine disruption in a marine amphipod? Field observations of intersexuality and de-masculinisation. Marine Environ Res. (2004) 58:169–73. 10.1016/j.marenvres.2004.03.013 15178030

[63] Takahashi T Araki A Nomura Y Koga M Arizono K The occurrence of dual-gender imposex in Japanese freshwater crab. J Health Sci. (2000) 46:376–9. 10.1248/jhs.46.376

[64] Khalaila I Manor R Weil S Granot Y Keller R Sagi A. The eyestalk-androgenic gland-testis endocrine axis in the crayfish *Cherax quadricarinatus*. Gen Comp Endocrinol. (2002) 127:147–56. 10.1016/S0016-6480(02)00031-X 12383442

[65] Rosen O Weil S Manor R Roth Z Khalaila I Sagi A. A crayfish insulin-like-binding protein: another piece in the androgenic gland insulin-like hormone puzzle is revealed. J Biol Chem. (2013) 288:22289–98. 10.1074/jbc.M113.484279 23775079 PMC3829320

[66] Chandler JC Aizen J Elizur A Hollander-Cohen L Battaglene SC Ventura T. Discovery of a novel insulin-like peptide and insulin binding proteins in the Eastern rock lobster *Sagmariasus verreauxi*. General Comparative Endocrinol. (2015) 215:76–87. 10.1016/j.ygcen.2014.08.018 25218129

[67] Aizen J Chandler JC Fitzgibbon QP Sagi A Battaglene SC Elizur A. Production of recombinant insulin-like androgenic gland hormones from three decapod species: *in vitro* testicular phosphorylation and activation of a newly identified tyrosine kinase receptor from the Eastern spiny lobster, *Sagmariasus verreauxi*. Gen Comp Endocrinol. (2016) 229:8–18. 10.1016/j.ygcen.2016.02.013 26883686

[68] Guo Q Li S Lv X Xiang J Sagi A Manor R. A putative insulin-like androgenic gland hormone receptor gene specifically expressed in male Chinese shrimp. Endocrinology. (2018) 159:2173–85. 10.1210/en.2017-03253 29596627

[69] Sroyraya M Chotwiwatthanakun C Stewart MJ Soonklang N Kornthong N Phoungpetchara I. Bilateral eyestalk ablation of the blue swimmer crab, Portunus pelagicus, produces hypertrophy of the androgenic gland and an increase of cells producing insulin-like androgenic gland hormone. Tissue Cell. (2010) 42:293–300. 10.1016/j.tice.2010.07.003 20817240

[70] Chung JS Manor R Sagi A. Cloning of an insulin-like androgenic gland factor (IAG) from the blue crab, *Callinectes sapidus*: implications for eyestalk regulation of IAG expression. Gen Comp Endocrinol. (2011) 173:4–10. 10.1016/j.ygcen.2011.04.017 21596044

[71] Rosen O Manor R Weil S Aflalo ED Bakhrat A Abdu U. An androgenic gland membrane-anchored gene associated with the crustacean insulin-like androgenic gland hormone. J Exp Biol. (2013) 216:2122–8. 10.1242/jeb.080523 23470660

[72] Keller R. Crustacean neuropeptides - structures, functions and comparative aspects. Experientia. (1992) 48:439–48. 10.1007/BF01928162 1601108

[73] Li F Bai H Zhang W Fu H Jiang F Liang G. Cloning of genomic sequences of three crustacean hyperglycemic hormone superfamily genes and elucidation of their roles of regulating insulin-like androgenic gland hormone gene. Gene. (2015) 561:68–75. 10.1016/j.gene.2015.02.012 25680292

[74] Kruangkum T Saetan J Chotwiwatthanakun C Vanichviriyakit R Thongrod S Thintharua P. Co-culture of males with late premolt to early postmolt female giant freshwater prawns, *Macrobrachium rosenbergii* resulted in greater abundances of insulin-like androgenic gland hormone and gonad maturation in male prawns as a result of olfactory receptors. Anim Reprod Sci. (2019) 210:106198. 10.1016/j.anireprosci.2019.106198 31635776

[75] Charniaux-Cotton H. Discovery in, an amphipod crustacean (*Orchestia gammarella*) of an endocrine gland responsible for the differentiation of primary and secondary male sex characteristics. C R Hebd Seances Acad Sci. (1954) 239:780–2. 13209865

[76] Katakura Y Transformation of ovary into testis following implantation of androgenous glands in *Armadillidium vulgare*, an isopod crustacean. Annot Zool Jpn. (1960) 33:241–4.

[77] Charniaux-Cotton H Contrôle hormonal de la différenciation du sexe et de la reproduction chez les Crustacés supérieurs. Bull Soc Zool Fr. (1958) 83:314–36.

[78] Charniaux-Cotton H Masculinisation des femelles de la crevette a hermaphrodisme proterandrique *Lysmata seticaudata*, par greffe de glandes androgenes. Interpretation de l'hermaphrodisme chez les Decapodes Comp Rend Acad Sci Paris. (1959) 249:1580–2.

[79] Berreur-Bonnenfant J Charniaux-Cotton H Hermaphrodisme protérandrique et fonctionnemente de la zone germinative chez la crevette *Pandalus borealis* Kröyer. Bull Soc Zool France. (1965) 90:259.

[80] Payen G Experiences de graffes de glandes androgenes sur la femelle pubere du Crabe *Rhithropanopeus harrisii* (Gould) (Crustace, Decapode). C R Hebd Seances Acad Sci. (1969) 268:393–6. 4975503

[81] Charniaux-Cotton H Cazes M Premiere masculinisation des femelles d'un Crustace Decapode gonochorique, *Palaemonetes varians* (Leach), par greffes de glandes androgenes. C R Acad Sci Paris. (1979) 288:1707–9.

[82] Frelon M Debenest C Martin G Masculinization of the ditch shrimp *Palaemonetes varians* (Leach, 1814). A re-evaluation using scanning electron microscopy (Decapoda, Caridea, Palaemonidae) Crustaceana. (1993) 105–10. 10.1163/156854093X00423

[83] Nagamine C Knight AW Maggenti A Paxman G. Effects of androgenic gland ablation on male primary and secondary sexual characteristics in the Malaysian prawn, *Macrobrachium rosenbergii* (de Man) (Decapoda, Palaemonidae), with first evidence of induced feminization in a nonhermaphroditic decapod. Gen Comp Endocrinol. (1980) 41:423–41. 10.1016/0016-6480(80)90048-9 7409450

[84] Nagamine C Knight AW Maggenti A Paxman G. Masculinization of female *Macrobrachium rosenbergii* (de Man) (Decapoda, Palaemonidae) by androgenic gland implantation. Gen Comp Endocrinol. (1980) 41:442–57. 10.1016/0016-6480(80)90049-0 7190950

[85] Aflalo ED Hoang TTT Nguyen VH Lam Q Nguyen DM Trinh QS A novel two-step procedure for mass production of all-male populations of the giant freshwater prawn *Macrobrachium rosenbergii*. Aquaculture. (2006) 256:468–78. 10.1016/j.aquaculture.2006.01.035

[86] Ventura T Manor R Aflalo ED Weil S Rosen O Sagi A. Timing sexual differentiation: full functional sex reversal achieved through silencing of a single insulin-like gene in the prawn, *Macrobrachium rosenbergii*. Biol Reprod. (2012) 86:1–6. 10.1095/biolreprod.111.097261 22133694

[87] Nagamine C Knight AW Masculinization of female crayfish, *Procambarus clarki* (Girard). Int J Invertebr Reprod Dev. (1987) 11:77–87. 10.1080/01688170.1987.10510268

[88] Taketomi Y Nishikawa S Implantation of androgenic glands into immature female crayfish, *Procambarus clarkii*, with masculinization of sexual characteristics. J Crustacean Biol. (1996) 16:232–9. 10.1163/193724096X00027

[89] Lee T Shigesawa R Yamazaki F Partial masculinization of female *Eriocheir japonicus* (Brachyura, Grapsidae) by androgenic gland implantation. Aquaculture Sci. (1993) 41:311–9.

[90] Fowler RJ Leonard BV The structure and function of the androgenic gland in *Cherax destructor* (Decapoda: Parastacidae). Aquaculture. (1999) 171:135–48. 10.1016/S0044-8486(98)00416-5

[91] Khalaila I Katz T Abdu U Yehezkel G Sagi A. Effects of implantation of hypertrophied androgenic glands on sexual characters and physiology of the reproductive system in the female red claw crayfish, *Cherax quadricarinatus*. Gen Comp Endocrinol. (2001) 121:242–9. 10.1006/gcen.2001.7607 11254366

[92] Sagi A Manor R Segall C Davis C Khalaila I On intersexuality in the crayfish *Cherax quadricarinatus*: an inducible sexual plasticity model. Invertebr Reprod Dev. (2002) 41:27–33. 10.1080/07924259.2002.9652732

[93] Barki A Karplus I Manor R Sagi A. Intersexuality and behavior in crayfish: the de-masculinization effects of androgenic gland ablation. Horm Behav. (2006) 50:322–31. 10.1016/j.yhbeh.2006.03.017 16769065

[94] Rosen O Manor R Weil S Gafni O Linial A Aflalo ED. A sexual shift induced by silencing of a single insulin-like gene in crayfish: ovarian upregulation and testicular degeneration. PLoS ONE. (2010) 5:e15281. 10.1371/journal.pone.0015281 21151555 PMC3000327

[95] Cui Z Liu H Lo TS Chu KH. Inhibitory effects of the androgenic gland on ovarian development in the mud crab *Scylla paramamosain*. Comp Biochem Physiol A Mol Integr Physiol. (2005) 140:343–8. 10.1016/j.cbpb.2005.01.017 15792600

[96] Liu H Cai SL Zhang CF Chu KH Masculinization of female *Eriocheir sinensis* by injecting the extract of androgenic gland of *E. sinensis* and *Scylla paramamosain*. J Fish China. (2006) 30:577–85.

[97] Kato M Hiruta C Tochinai S. Androgenic gland implantation induces partial masculinization in marmorkrebs *Procambarus fallax* f. virginalis. Zool Sci. (2015) 32:459–64. 10.2108/zs150028 26428724

[98] Alfaro-Montoya J Hernández-Noguera L Vega-Alpízar L Umaña-Castro R Effects of androgenic gland ablation on growth, sexual characters and spermatogenesis of the white shrimp, *Litopenaeus vannamei* (Decapoda: Penaeidae) males. Aquac Res. (2016) 47:2768–77. 10.1111/are.12727

[99] Vega-Alpízar JL Alfaro-Montoya J Hernández-Noguera L Umaña-Castro R Aflalo ED Sagi A Implant recognition and gender expression following ampoule-androgenic gland implantation in *Litopenaeus vannamei* females (Penaeidae). Aquaculture. (2017) 468:471–80. 10.1016/j.aquaculture.2016.11.007

[100] Ford AT. Can you feminise a crustacean? Aquatic Toxicol. (2008) 88:316–21. 10.1016/j.aquatox.2008.04.013 18550186

[101] Charniaux-Cotton H Payen G Sexual differentiation. In: eds. Bliss DE Mantel LH The Biology of Crustacea. New York, NY: Academic Press, (1985) 217–99. 10.1016/B978-0-12-106409-9.50015-4

[102] Sagi A Khalaila I Barki A Hulata G Karplus I. Intersex red claw crayfish, *Cherax quadricarinatus* (von Martens): functional males with pre-vitellogenic ovaries. Biol Bull. (1996) 190:16–23. 10.2307/1542672 29244551

[103] Scholtz G Braband A Tolley L Reimann A Mittmann B Lukhaup C. Ecology: parthenogenesis in an outsider crayfish. Nature. (2003) 421:806–8. 10.1038/421806a 12594502

[104] Martin P Kohlmann K Scholtz G. The parthenogenetic Marmorkrebs (marbled crayfish) produces genetically uniform offspring. Naturwissenschaften. (2007) 94:843–6. 10.1007/s00114-007-0260-0 17541537

[105] Levy T Rosen O Simons O Alkalay AS Sagi A The gene encoding the insulin-like androgenic gland hormone in an all-female parthenogenetic crayfish. PLoS ONE. (2017) 12:e0189982 10.1371/journal.pone.0189982 29261765 PMC5738133

[106] Martin P Dorn NJ Kawai T van der Heiden C Scholtz G The enigmatic Marmorkrebs (marbled crayfish) is the parthenogenetic form of *Procambarus fallax* (Hagen, 1870). Contrib Zool. (2010) 79:107–18. 10.1163/18759866-07903003

[107] Lezer Y Aflalo ED Manor R Sharabi O Abilevich LK Sagi A On the safety of RNAi usage in aquaculture: the case of all-male prawn stocks generated through manipulation of the insulin-like androgenic gland hormone. Aquaculture. (2015) 435:157–66. 10.1016/j.aquaculture.2014.09.040

[108] Heasman J. Morpholino oligos: making sense of antisense? Dev Biol. (2002) 243:209–14. 10.1006/dbio.2001.0565 11884031

[109] Doudna JA Charpentier E. The new frontier of genome engineering with CRISPR-Cas9. Science. (2014) 346:1077. 10.1126/science.1258096 25430774

[110] Hunter GA Donaldson EM Stoss J Baker I Production of monosex female groups of chinook salmon (*Oncorhynchus tshawytscha*) by the fertilization of normal ova with sperm from sex-reversed females. Aquaculture. (1983) 33:355–64. 10.1016/0044-8486(83)90414-3

[111] Pongthana N Penman DJ Baoprasertkul P Hussain MG Islam MS Powell SF Monosex female production in the silver barb (*Puntius gonionotus* Bleeker). Aquaculture. (1999) 173:247–56. 10.1016/S0044-8486(98)00449-9

[112] Beardmore JA Mair GC Lewis RI Monosex male production in finfish as exemplified by tilapia: applications, problems, and prospects. Aquaculture. (2001) 197:283–301. 10.1016/B978-0-444-50913-0.50015-1

[113] Meyer A Cloete SWP Brown CR The influence of separate-sex rearing on ostrich behaviour and skin damage. S Afr J Anim Sci. (2003) 33:95–104. 10.4314/sajas.v33i2.3762

[114] Kul S Seker I Yildirim O Effect of separate and mixed rearing according to sex on fattening performance and carcass characteristics in Japanese quails (*Coturnix coturnix Japonica*). Archiv Fur Tierzucht-Arch Animal Breeding. (2006) 49:607–14. 10.5194/aab-49-607-2006

[115] Krautwald-Junghanns ME Cramer K Fischer B Forster A Galli R Kremer F. Current approaches to avoid the culling of day-old male chicks in the layer industry, with special reference to spectroscopic methods. Poult Sci. (2018) 97:749–57. 10.3382/ps/pex389 29294120

[116] Leneindre P Influence of rearing conditions and breed on social-behavior and activity of cattle in novel environments. Appl Anim Behav Sci. (1989) 23:129–40. 10.1016/0168-1591(89)90013-0

[117] Sagi A Raanan Z Cohen D Wax Y Production of *Macrobrachium rosenbergii* in monosex populations - yield characteristics under intensive monoculture conditions in cages. Aquaculture. (1986) 51:265–75. 10.1016/0044-8486(86)90318-2

[118] Hansford SW Hewitt DR Growth and nutrient digestibility by male and female *Penaeus monodon*- evidence of sexual dimorphism. Aquaculture. (1994) 125:147–54. 10.1016/0044-8486(94)90291-7

[119] Ventura T Sagi A. The insulin-like androgenic gland hormone in crustaceans: from a single gene silencing to a wide array of sexual manipulation-based biotechnologies. Biotechnol Adv. (2012) 30:1543–50. 10.1016/j.biotechadv.2012.04.008 22561950

[120] Sagi A Aflalo ED The androgenic gland and monosex culture of freshwater prawn *Macrobrachium rosenbergii* (De Man): a biotechnological perspective. Aquac Res. (2005) 36:231–7. 10.1111/j.1365-2109.2005.01238.x

[121] Moss DR Hennig OL Moss SM Sexual growth dimorphism in penaeid shrimp. Potential for all female culture? Global Aquaculture Avdocate. (2002) 5:60–1.

[122] Moss DR Moss SM Effects of gender and size on feed acquisition in the Pacific white shrimp *Litopenaeus vannamei*. J World Aquac Soc. (2006) 37:161–7. 10.1111/j.1749-7345.2006.00022.x

[123] Schneiker J Weisser WW Settele J Nguyen VS Bustamante JV Marquez L Is there hope for sustainable management of golden apple snails, a major invasive pest in irrigated rice? NJAS-Wageningen. J Life Sci. (2016) 79:11–21. 10.1016/j.njas.2016.07.001

[124] Alkalay-Savaya A Rosen O Sokolow SH Faye YPW Fzye DS Aflalo ED The prawn *Macrobrachium vollenhovenii* in the Senegal River basin: towards sustainable restocking of all-male populations for biological control of schistosomiasis. PLoS Negl Trop Dis. (2014) 8:e3060 10.1371/journal.pntd.0003060 25166746 PMC4148216

[125] Sokolow SH Wood CL Jones IJ Swartz SJ Lopez M Hsieh MH. Global assessment of schistosomiasis control over the past century shows targeting the snail intermediate host works best. PLoS Negl Trop Dis. (2016) 10:e0004794. 10.1371/journal.pntd.0004794 27441556 PMC4956325

[126] Sokolow SH Jones IJ Jocque M La D Cords O Knight A. Nearly 400 million people are at higher risk of schistosomiasis because dams block the migration of snail-eating river prawns. Philos Trans R Soc B Biol Sci. (2017) 372:20160127. 10.1098/rstb.2016.0127 28438916 PMC5413875

[127] Hoover CM Sokolow SH Kemp J Sanchirico JN Lund AJ Jones IJ Modelled effects of prawn aquaculture on poverty alleviation and schistosomiasis control. Nature Sustainability. (2019) 2:611–20. 10.1038/s41893-019-0301-7 PMC7731924 33313425

[128] Savaya A Glassner H Livne-Luzon S Chishinski R Molcho J Aflalo ED Prawn monosex populations as biocontrol agents for snail vectors of fish parasites. Aquaculture. (2020) 520:735016 10.1016/j.aquaculture.2020.735016

[129] Van Lenteren JC Babendreier D Bigler F Burgio G Hokkanen HMT Kuske S Environmental risk assessment of exotic natural enemies used in inundative biological control. BioControl. (2003) 48:3–38. 10.1023/A:1021262931608

[130] Curtis MC Jones CM Observations on monosex culture of redclaw crayfish *Cherax quadricarinatus* von Martens (Decapoda: Parastacidae) in earthen ponds. J World Aquac Soc. (1995) 26:154–9. 10.1111/j.1749-7345.1995.tb00238.x

[131] Lawrence CS Cheng YW Morrissy NM Williams IH A comparison of mixed-sex vs. monosex growout and different diets on the growth rate of freshwater crayfish (Cherax albidus). Aquaculture. (2000) 185:281–9. 10.1016/S0044-8486(99)00358-0

[132] Rodgers LJ Saoud PI Rouse DB The effects of monosex culture and stocking density on survival, growth and yield of redclaw crayfish (*Cherax quadricarinatus*) in earthen ponds. Aquaculture. (2006) 259:164–8. 10.1016/j.aquaculture.2005.11.056

[133] Jong KJ Growth of the spiny lobster *panulirus homarus* (linnaeus, 1758), depending on sex and influenced by reproduction (decapoda, palinuridae). Crustaceana. (1993) 64:18–23. 10.1163/156854093X00027

[134] Siddiqui AQ Al-Hafedh YS Al-Harbi AH Ali SA Effects of stocking density and monosex culture of freshwater prawn Macrobrachium rosenbergii on growth and production in concrete tanks in Saudi Arabia. J World Aquac Soc. (1997) 28:106–12. 10.1111/j.1749-7345.1997.tb00968.x

[135] Trino AT Millamena OM Keenan C Commercial evaluation of monosex pond culture of the mud crab *Scylla* species at three stocking densities in the Philippines. Aquaculture. (1999) 174:109–18. 10.1016/S0044-8486(99)00002-2

[136] Argue BJ Arce SM Lotz JM Moss SM Selective breeding of Pacific white shrimp (*Litopenaeus vannamei*) for growth and resistance to Taura Syndrome Virus. Aquaculture. (2002) 204:447–60. 10.1016/S0044-8486(01)00830-4

[137] Wu X Cheng Y Sui L Yang X Nan T Wang J Biochemical composition of pond-reared and lake-stocked Chinese mitten crab *Eriocheir sinensis* (H. *Milne-Edwards*) broodstock. Aquaculture Res. (2007) 38:1459–67. 10.1111/j.1365-2109.2007.01728.x

[138] Malecha S Mather PB Hurwood D Genetics. In: New MB Valenti WC Tidwell JH D'Abramo LR Kutty MN editors. Freshwater Prawns: Biology and Farming. Chichester, UK: Wiley-Blackwell (2010). p. 278–315. 10.1002/9781444314649.ch15

[139] Otoshi CA Arce SM Moss SM Growth and reproductive performance of broodstock shrimp reared in a biosecure recirculating aquaculture system versus a flow-through pond. Aquac Eng. (2003) 29:93–107. 10.1016/S0144-8609(03)00048-7

[140] Gopal C Gopikrishna G Krishna G Jahageerdar SS Rye M Hayes B Weight and time of onset of female-superior sexual dimorphism in pond reared *Penaeus monodon*. Aquaculture. (2010) 300:237–9. 10.1016/j.aquaculture.2010.01.007

[141] Malecha S The case for all-female freshwater prawn, *Macrobrachium rosenbergii* (De Man), culture. Aquac Res. (2012) 43:1038–48. 10.1111/j.1365-2109.2011.03007.x

[142] Nair CM Salin KR Raju MS Sebastian M Economic analysis of monosex culture of giant freshwater prawn (*Macrobrachium rosenbergii* De Man): a case study. Aquac Res. (2006) 37:949–54. 10.1111/j.1365-2109.2006.01521.x

[143] Shpak N Manor R Aflalo ED Sagi A Three generations of cultured prawn without W chromosome. Aquaculture. (2017) 467:41–8. 10.1016/j.aquaculture.2016.06.008

[144] Molcho J Levy T Benet A Naor A Savaya A Manor R Three generations of prawns without the Z chromosome: Viable WW *Macrobrachium rosenbergii* all-female populations in polyculture with *Oreochromis niloticus*. Aquaculture. (2020) 515:734531 10.1016/j.aquaculture.2019.734531

[145] Phoenix CH Goy RW Gerall AA Young WC. Organizing action of prenatally administered testosterone propionate on the tissues mediating mating behavior in the female guinea pig. Endocrinology. (1959) 65:369–82. 10.1210/endo-65-3-369 14432658

[146] Breedlove SM Arnold AP. Hormonal control of a developing neuromuscular system. II Sensitive periods for the androgen-induced masculinization of the rat spinal nucleus of the bulbocavernosus. J Neurosci. (1983) 3:424–32. 10.1523/JNEUROSCI.03-02-00424.1983 6822871 PMC6564495

[147] Noriega NC Evolutionary perspectives on sex steroids in the vertebrates. In: Kahn SM editors. Sex Steroids. Rijeka: InTech, (2012) 3–34.

[148] Hunter GA Donaldson EM Hormonal sex control and its application to fish culture. In: Hoar WS Randall DJ Donaldson EM editors. Fish Physiology. New York, NY: Academic Press, (1983) 223–303. 10.1016/S1546-5098(08)60305-2

[149] Pandian TJ Sheela SG Hormonal induction of sex reversal in fish. Aquaculture. (1995) 138:1–22. 10.1016/0044-8486(95)01075-0

[150] Sayed AEH Moneeb RH Hematological and biochemical characters of monosex tilapia (*Oreochromis niloticus*, Linnaeus, 1758) cultivated using methyltestosterone. J Basic Appl Zool. (2015) 72:36–42. 10.1016/j.jobaz.2015.03.002

[151] Lafont R Mathieu M. Steroids in aquatic invertebrates. Ecotoxicology. (2007) 16:109–30. 10.1007/s10646-006-0113-1 17238002

[152] Gottfried H Lusis O. Steroids of invertebrates: the *in vitro* production of II-ketotestosterone and other steroids by the eggs of the slug, *Arion ater rufus* (Linn.). Nature. (1966) 212:1488–9. 10.1038/2121488a0 21090426

[153] Gottfried H Dorfman RI Wall PE Steroids of invertebrates: production of oestrogens by an accessory reproductive tissue of the slug Arion ater rufus (Linn). Nature. (1967) 215:409–10. 10.1038/215409a0 6058299

[154] Reis-Henriques MA Le Guellec D Remy-Martin JP Adessi GL Studies of endogenous steroids from the marine mollusc *Mytilus edulis* L. by gas chromatography and mass spectrometry. Comp Biochem Physiol B. (1990) 95:303–9. 10.1016/0305-0491(90)90080-D

[155] Siah A Pellerin J Benosman A Gagne JP Amiard JC. Seasonal gonad progesterone pattern in the soft-shell clam *Mya arenaria*. Comp Biochem Physiol A. (2002) 132:499–511. 10.1016/S1095-6433(02)00095-8 12020666

[156] Di Cosmo A Paolucci M Di Cristo C Botte V Ciarcia G. Progesterone receptor in the reproductive system of the female of *Octopus vulgaris*: characterization and immunolocalization. Mol Reprod Dev. (1998) 50:451–60. 10.1002/(SICI)1098-2795(199808)50:4<451::AID-MRD9>3.0.CO;2-H 9669529

[157] Di Cosmo A Di Cristo C Paolucci M. Sex steroid hormone fluctuations and morphological changes of the reproductive system of the female of *Octopus vulgaris* throughout the annual cycle. J Exp Zool. (2001) 289:33–47. 10.1002/1097-010X(20010101/31)289:1<33::AID-JEZ4>3.0.CO;2-A 11169491

[158] Di Cosmo A Di Cristo C Paolucci M. A estradiol-17beta receptor in the reproductive system of the female of *Octopus vulgaris*: characterization and immunolocalization. Mol Reprod Dev. (2002) 61:367–75. 10.1002/mrd.10014 11835582

[159] Donahue JK Jennings ED The occurrence of estrogenic substances in the ovaries of echinoderms. Endocrinology. (1937) 21:690–1. 10.1210/endo-21-5-690

[160] Botticelli CR Hisaw FLJr Wotiz HH. Estradiol-17beta and progesterone in ovaries of starfish (*Pisaster ochraceous*). Proc Soc Exp Biol Med. (1960) 103:875–7. 10.3181/00379727-103-25704 13803144

[161] Botticelli CR Hisaw FL Jr Wotiz HH Estrogens and progesterone in the sea urchin (*Strongylocentrotus franciscanus*) and pecten (*Pecten hericius*). Proc Soc Exp Biol Med. (1961) 106:887–9. 10.3181/00379727-106-26511

[162] Schallig HDFH Glasmeier A de Jong-Brink M Vertebrate-type steroids in cercariae of the schistosome *Trichobilharzia ocellata*. Parasitol Res. (1992) 78:709–11. 10.1007/BF00931527

[163] Mouneyrac C Pellerin J Moukrim A Alla AA Durou C Viault N. *In situ* relationship between energy reserves and steroid hormone levels in *Nereis diversicolor* (OF Müller) from clean and contaminated sites. Ecotoxicol Environ Saf. (2006) 65:181–7. 10.1016/j.ecoenv.2005.07.002 16157376

[164] Kohler HR Kloas W Schirling M Lutz I Reye AL Langen JS. Sex steroid receptor evolution and signalling in aquatic invertebrates. Ecotoxicology. (2007) 16:131–43. 10.1007/s10646-006-0111-3 17219085

[165] Atkinson S Atkinson MJ Detection of estradiol-17β during a mass coral spawn. Coral Reefs. (1992) 11:33–5. 10.1007/BF00291932

[166] Irwin JC Utian WH Eckert RL. Sex steroids and growth factors differentially regulate the growth and differentiation of cultured human endometrial stromal cells. Endocrinology. (1991) 129:2385–92. 10.1210/endo-129-5-2385 1935772

[167] Chang ES Mykles DL. Regulation of crustacean molting: a review and our perspectives. Gen Comp Endocrinol. (2011) 172:323–30. 10.1016/j.ygcen.2011.04.003 21501612

[168] Passano LM Jyssum S. The role of the Y-organ in crab proecdysis and limb regeneration. Comp Biochem Physiol. (1963) 9:195–213. 10.1016/0010-406X(63)90044-6 14111887

[169] Gersch M Eibisch H Bohm GA Koolman J. Ecdysteroid production by the cephalic gland of the crayfish *Orconectes limosus*. Gen Comp Endocrinol. (1979) 39:505–11. 10.1016/0016-6480(79)90238-7 520813

[170] Shyamal S Das S Guruacharya A Mykles DL Durica DS. Transcriptomic analysis of crustacean molting gland (Y-organ) regulation via the mTOR signaling pathway. Sci Rep. (2018) 8:7307. 10.1038/s41598-018-25368-x 29743490 PMC5943448

[171] Bergstrom BI The biology of *Pandalus*. Adv Mar Biol. (2000) 38:55–245. 10.1016/S0065-2881(00)38003-8

[172] Yano I Induced ovarian maturation and spawning in greasyback shrimp, *Metapenaeus ensis*, by progesterone. Aquaculture. (1985) 47:223–9. 10.1016/0044-8486(85)90068-7

[173] Yano I Effect of 17α-hydroxy-progesterone on vitellogenin secretion in kuruma prawn, *Penaeus japonicus*. Aquaculture. (1987) 61:49–57. 10.1016/0044-8486(87)90337-1

[174] Liu M Pan J Liu Z Cheng Y Gong J Wu X Effect of estradiol on vitellogenesis and oocyte development of female swimming crab, *Portunus trituberculatus*. Aquaculture. (2018) 486:240–5. 10.1016/j.aquaculture.2017.12.034

[175] Sarojini S Comparison of the effects of androgenic hormone and testosterone propionate on the female ocypod crab. Curr Sci. (1963) 32:411–2.

[176] Nagabhushanam R Kulkarni GK Effect of exogenous testosterone on the androgenic gland and testis of a marine penaeid prawn, *Parapenaeopsis hardwickii* (Miers) (Crustacea, Decapoda, Penaeidae). Aquaculture. (1981) 23:19–27. 10.1016/0044-8486(81)90004-1

[177] Sugestya ING Widodo MS Soeprijanto A Effect of 17β-estradiol on feminization, growth rate and survival rate of pacific white shrimp (*Litopenaeus vannamei*, Boone 1931) postlarvae. J Exp Life Sci. (2018) 8:37–42. 10.21776/ub.jels.2018.008.01.06

[178] Cherif-Feildel M Heude Berthelin C Adeline B Riviere G Favrel P Kellner K. Molecular evolution and functional characterisation of insulin related peptides in molluscs: contributions of *Crassostrea gigas* genomic and transcriptomic-wide screening. Gen Comp Endocrinol. (2019) 271:15–29. 10.1016/j.ygcen.2018.10.019 30389328

[179] Le Roith D Shiloach J Roth J Lesniak MA. Evolutionary origins of vertebrate hormones: substances similar to mammalian insulins are native to unicellular eukaryotes. Proc Natl Acad Sci USA. (1980) 77:6184–8. 10.1073/pnas.77.10.6184 6449704 PMC350239

[180] McRory JE Sherwood NM. Ancient divergence of insulin and insulin-like growth factor. DNA Cell Biol. (1997) 16:939–49. 10.1089/dna.1997.16.939 9303436

[181] Shabanpoor F Separovic F Wade JD. The human insulin superfamily of polypeptide hormones. Vitam Horm. (2009) 80:1–31. 10.1016/S0083-6729(08)00601-8 19251032

[182] Daughaday WH Rotwein P. Insulin-like growth factors I and II. Peptide, messenger ribonucleic acid and gene structures, serum, and tissue concentrations. Endocrine Rev. (1989) 10:68–91. 10.1210/edrv-10-1-68 2666112

[183] Jones JI Clemmons DR. Insulin-like growth factors and their binding proteins: biological actions. Endocr Rev. (1995) 16:3–34. 10.1210/er.16.1.3 7758431

[184] Conklin D Lofton-Day CE Haldeman BA Ching A Whitmore TE Lok S. Identification of INSL5, a new member of the insulin superfamily. Genomics. (1999) 60:50–6. 10.1006/geno.1999.5899 10458910

[185] Sliwowska JH Fergani C Gawalek M Skowronska B Fichna P Lehman MN. Insulin: its role in the central control of reproduction. Physiol Behav. (2014) 133:197–206. 10.1016/j.physbeh.2014.05.021 24874777 PMC4084551

[186] Collip JB The demonstration of an insulin-like substance in the tissues of the clam (*Mya arenaria*). J Biol Chem. (1923) 55:16.

[187] Veenstra JA. Neurohormones and neuropeptides encoded by the genome of *Lottia gigantea*, with reference to other mollusks and insects. Gen Comp Endocrinol. (2010) 167:86–103. 10.1016/j.ygcen.2010.02.010 20171220

[188] LeRoith D Lesniak MA Roth J. Insulin in insects and annelids. Diabetes. (1981) 30:70–6. 10.2337/diabetes.30.1.70 6785127

[189] Wang S Luo X Zhang S Yin C Dou Y Cai X. Identification of putative insulin-like peptides and components of insulin signaling pathways in parasitic platyhelminths by the use of genome-wide screening. FEBS J. (2014) 281:877–93. 10.1111/febs.12655 24286276

[190] Anctil M. Chemical transmission in the sea anemone *Nematostella vectensis*: a genomic perspective. Comp Biochem Physiol. (2009) 4:268–89. 10.1016/j.cbd.2009.07.001 20403752

[191] Robitzki A Schroder HC Ugarkovic D Pfeifer K Uhlenbruck G Muller WE. Demonstration of an endocrine signaling circuit for insulin in the sponge *Geodia cydonium*. EMBO J. (1989) 8:2905–9. 10.1002/j.1460-2075.1989.tb08439.x 2531072 PMC401354

[192] Pierce SB Costa M Wisotzkey R Devadhar S Homburger SA Buchman AR. Regulation of DAF-2 receptor signaling by human insulin and ins-1, a member of the unusually large and diverse C. elegans insulin gene family. Genes Dev. (2001) 15:672–86. 10.1101/gad.867301 11274053 PMC312654

[193] Yoshida I Moto K Sakurai S Iwami M. A novel member of the bombyxin gene family: structure and expression of bombyxin G1 gene, an insulin-related peptide gene of the silkmoth *Bombyx mori*. Dev Genes Evol. (1998) 208:407–10. 10.1007/s004270050197 9732555

[194] Sauman I Reppert SM. Molecular characterization of prothoracicotropic hormone (PTTH) from the giant silkmoth *Antheraea pernyi*: developmental appearance of PTTH-expressing cells and relationship to circadian clock cells in central brain. Dev Biol. (1996) 178:418–29. 10.1006/dbio.1996.0228 8812139

[195] Nagasawa H Kataoka H Isogai A Tamura S Suzuki A Ishizaki H. Amino-terminal amino acid sequence of the silkworm prothoracicotropic hormone: homology with insulin. Science. (1984) 226:1344–5. 10.1126/science.226.4680.1344 17832633

[196] Nijhout HF Grunert LW. Bombyxin is a growth factor for wing imaginal disks in Lepidoptera. Proc Natl Acad Sci USA. (2002) 99:15446–50. 10.1073/pnas.242548399 12429853 PMC137736

[197] Goto S Loeb MJ Takeda M. Bombyxin stimulates proliferation of cultured stem cells derived from *Heliothis virescens* and *Mamestra brassicae* larvae1. In Vitro Cell Dev Biol Anim. (2005) 41:38–42. 10.1290/0312092.1 15926858

[198] Maniere G Rondot I Bullesbach EE Gautron F Vanhems E Delbecque JP. Control of ovarian steroidogenesis by insulin-like peptides in the blowfly (*Phormia regina*). J Endocrinol. (2004) 181:147–56. 10.1677/joe.0.1810147 15072575

[199] Kromer-Metzger E Lagueux M. Expression of the gene encoding an insulin-related peptide in *Locusta* (Insecta, Orthoptera). Evidence for alternative promoter usage. Eur J Biochem. (1994) 221:427–34. 10.1111/j.1432-1033.1994.tb18755.x 8168530

[200] Meneses P De Los Angeles Ortiz M. A protein extract from *Drosophila melanogaster* with insulin-like activity. Compar Biochem Physiol A. (1975) 51:483–5. 10.1016/0300-9629(75)90398-9 237670

[201] Yamaguchi T Fernandez R Roth RA. Comparison of the signaling abilities of the *Drosophila* and human insulin receptors in mammalian cells. Biochemistry. (1995) 34:4962–8. 10.1021/bi00015a007 7711018

[202] Gutierrez A Nieto J Pozo F Stern S Schoofs L. Effect of insulin/IGF-I like peptides on glucose metabolism in the white shrimp *Penaeus vannamei*. Gen Comp Endocrinol. (2007) 153:170–5. 10.1016/j.ygcen.2007.04.014 17574553

[203] Wang L Chen H Wang L Song L. An insulin-like peptide serves as a regulator of glucose metabolism in the immune response of Chinese mitten crab *Eriocheir sinensis*. Dev Comp Immunol. (2020) 108:103686. 10.1016/j.dci.2020.103686 32205179

